# Engineering *Bacillus megaterium* for production of functional intracellular materials

**DOI:** 10.1186/s12934-017-0823-5

**Published:** 2017-11-22

**Authors:** Katrin Grage, Paul McDermott, Bernd H. A. Rehm

**Affiliations:** 1 Institute of Fundamental Sciences, Massey University, Private Bag 11222, Palmerston North, 4442 New Zealand; 2 Bioline Reagents Ltd., Unit 16, The Edge Business Centre, Humber Road, London, NW2 6EW UK; 3 Centre for Cell Factories and Biopolymers, Griffith Institute for Drug Discovery, Griffith University, Don Young Road, Nathan, QLD Australia

**Keywords:** Poly(3-hydroxybutyrate) (PHB), PHA synthase, *Bacillus megaterium*, Endotoxin, Genetic engineering, Functionalized beads, ZZ-domain, IgG binding, Sporulation, ∆*spoIIE*

## Abstract

**Background:**

Over the last 10–15 years, a technology has been developed to engineer bacterial poly(3-hydroxybutyrate) (PHB) inclusions as functionalized beads, for applications such as vaccines, diagnostics and enzyme immobilization. This has been achieved by translational fusion of foreign proteins to the PHB synthase (PhaC). The respective fusion protein mediates self-assembly of PHB inclusions displaying the desired protein function. So far, beads have mainly been produced in recombinant *Escherichia coli,* which is problematic for some applications as the lipopolysaccharides (LPS) co-purified with such inclusions are toxic to humans and animals.

**Results:**

In this study, we have bioengineered the formation of functional PHB inclusions in the Gram-positive bacterium *Bacillus megaterium*, an LPS-free and established industrial production host. As *B. megaterium* is a natural PHB producer, the PHB-negative strain PHA05 was used to avoid any background PHB production. Plasmid-mediated T7 promoter-driven expression of the genes encoding β-ketothiolase (*phaA*), acetoacetyl-CoA-reductase (*phaB*) and PHB synthase (*phaC*) enabled PHB production in *B. megaterium* PHA05. To produce functionalized PHB inclusions, the N- and C-terminus of PhaC was fused to four and two IgG binding Z-domains from *Staphylococcus aureus,* respectively. The ZZ-domain PhaC fusion protein was strongly overproduced at the surface of the PHB inclusions and the corresponding isolated ZZ-domain displaying PHB beads were found to purify IgG with a binding capacity of 40–50 mg IgG/g beads. As *B. megaterium* has the ability to sporulate and respective endospores could co-purify with cellular inclusions, a sporulation negative production strain was generated by disrupting the *spoIIE* gene in PHA05. This strain did not produce spores when tested under sporulation inducing conditions and it was still able to synthesize ZZ-domain displaying PHB beads.

**Conclusions:**

This study provides proof of concept for the successful genetic engineering of *B. megaterium* as a host for the production of functionalized PHB beads. Disruption of the *spoIIE* gene rendered *B. megaterium* incapable of sporulation but particularly suitable for production of functionalized PHB beads. This sporulation-negative mutant represents an improved industrial production strain for biotechnological processes otherwise impaired by the possibility of endospore formation.

**Electronic supplementary material:**

The online version of this article (10.1186/s12934-017-0823-5) contains supplementary material, which is available to authorized users.

## Background

Bacteria have been engineered to produce protein or polymer inclusions in order to efficiently recover proteins of interest or to immobilize and/or display various protein functions for medical and industrial uses [1–7]. Bacterial inclusions made of the biopolyester, poly(3-hydroxybutyrate) (PHB), have been extensively redesigned using bioengineering and synthetic biology approaches in order to obtain surface functionalized nano-/micro-spheres. Polyhydroxyalkanoates (PHAs) in general serve as carbon and energy storage. PHB is synthesized from the central metabolite acetyl-CoA, requiring consecutive conversions catalysed by three enzymes. The last PHB synthesis reaction is catalysed by the PHB Synthase (PhaC) which remains covalently bound to the emerging PHB granule [8, 9]. The unique underlying self-assembly pathway has been harnessed by translationally fusing proteins of interest to either terminus of PhaC, which ultimately enabled production of PHB beads displaying various protein functions [10–16]. This resulted in the development of a platform technology for the production of functionalized biobeads where a protein of interest is displayed at high density and homogenous orientation on the bead surface anchored via PhaC [10–13, 16–19]. Possible applications include diagnostics, affinity bioseparation and vaccine delivery and have been extensively and recently reviewed [20–22]. PolyBatics Ltd is using this technology to develop and produce a vaccine against tuberculosis (TB) and a diagnostic reagent for the detection of bovine TB [23, 24]. Most of these bead prototypes were produced in engineered *Escherichia coli*. Evidently, for medical applications, the use of *E. coli* as a production host can be problematic, as the lipopolysaccharides (LPS) synthesized by Gram-negative bacteria have an endotoxic effect in humans. The removal of these endotoxins during the purification of the PHB beads is considered to be laborious and costly. Gram-positive bacteria are a promising alternative as they do not contain LPS. Hence there is considerable interest to explore these as producers of various PHAs, both by screening for new strains as well as by optimizing established systems [25–30]. However, these efforts almost exclusively focus on exploiting the organism’s natural ability to generate PHAs and the production of PHAs as alternative (bulk) plastics. An attempt had previously been made to engineer *Lactococcus lactis* for the recombinant production of PHB beads, including functionalized beads displaying the IgG binding domain of *Staphylococcus aureus* protein A (ZZ-domain) [16, 31]. Functional beads had been obtained but overall PHB yields had been very low suggesting that the metabolism of *L. lactis* is probably not ideal for an engineered PHB production pathway. *Bacillus megaterium*, on the other hand, naturally produces PHB [29]. It is in fact the organism for which the accumulation of PHB was first reported in 1926 [32]. Other advantages of this organism include its large cell size (up to 1.5 × 4 μm) and the availability of overexpression systems with strong promoters [33–35]. For decades *B. megaterium* has been an important industrial workhorse for the production of enzymes, vitamins and drugs, and has more recently also been considered for the production of recombinant proteins [36–40]. In this study, we were aiming at engineering *B. megaterium* for recombinant production of PHB towards the assembly of functionalised PHB beads (ZZ beads). To avoid a background PHB synthesis, the PHB-negative strain PHA05 was used as a host. In this strain, the PHB synthase encoding genes had been deleted [41]. Furthermore, as *B. megaterium* is capable of sporulation [40], there is a risk that, especially if grown to high cell densities and under unfavourable conditions, endospores are formed. In order to avoid the presence of endospores in suspensions of purified biobeads, we investigated the possibility to engineer a sporulation-negative PHA05 strain.

## Results

### Bioengineering of *B. megaterium* for production of ZZ-domain displaying PHB beads

The genes *phaA*, *phaB* and *phaC* from *Cupriavidus necator* plus the DNA sequences encoding the N- and C-terminally fused Z-domains were synthesized (GenScript) as codon optimized for expression in *B. megaterium*. Genes and DNA fragments were inserted into vectors pMM1522, p1623hp and pPT7, respectively, in order to mediate PHB bead production in *B. megaterium* (Additional file 1: Figure S1). In pMM1522 and p1623hp, genes are expressed under the control of the xylose-inducible *xylA* promoter [34, 35]. Plasmid p1623hp is a derivative of pMM1522 with an improved promoter region and ribosome binding site. In pPT7 genes are expressed under the control of a T7 promoter, and a T7 RNA polymerase, whose expression is controlled by a xylose-inducible *xylA* promoter, is encoded by the plasmid pT7RNAP [33]. For both pMM1522 and pPT7, a control plasmid was constructed containing only *phaA* and *phaB*, and one containing *phaC*, *phaA* and *phaB* (in this order) to demonstrate production of non-functionalised PHB beads. To mediate production of functional PHB beads, plasmids containing ZZ/ZZ*phaC*-ZZ (encoding PhaC with four N-terminal and two C-terminal Z domains), *phaA* and *phaB* were made for all three systems. Genes *phaA* and *phaB* encode the enzymes β-ketothiolase and acetoacetyl-CoA-reductase and are required to convert acetyl-CoA into hydroxybutyryl-CoA, the substrate of PhaC [42]. *B. megaterium* PHA05 was transformed with all plasmids by protoplast transformation.

### Optimization of growth medium, growth and induction conditions

In recombinant *E. coli*, PHB beads have routinely been produced by growing production strains in glucose-supplemented LB medium. However, growth was unsatisfactory for *B. megaterium* PHA05 harbouring either of the new production plasmids when grown in LB supplemented with various carbon sources (Additional file 1: Table S1). The semi-defined A5 medium has previously been used to cultivate *B. megaterium* [39, 43]. Growth and PHB production were assessed for the three production systems in A5 medium supplemented with one of the following carbon sources: glucose, glycerol, fructose or pyruvate. Glucose-supplemented A5 medium and the T7 system were chosen for further studies as the T7 system seemed to result in the highest PHB production using the various carbon sources (as visually assessed by fluorescence microscopy of Nile Red stained cells) and glucose addition resulted in the highest OD_600_.(Additional file 1: Table S2). Recombinant *E. coli* is routinely cultivated for 48 h for PHB bead production. However, in the case of *B. megaterium*, measurements of cell density (OD_600_) and PHB content (GC/MS) indicated that both parameters had not increased any further after 48 h compared to 24 h, but instead seemed to decrease, so a standard cultivation time of 24 h was chosen (Additional file 1: Table S3). In order to optimize induction conditions, the amount of xylose and the time point of xylose addition were also varied. Doubling the amount of xylose added or adding xylose again after the initial induction seemed to have a slight positive effect of protein overproduction (Additional file 1: Figure S2) and on PHB production (Additional file 1: Table S4), whereas adding the inducer at a higher OD or increasing the glucose concentration seemed to be less effective. Subsequent cultures were therefore induced by the addition of xylose to a final concentration of 1% (w/v) at an OD_600_ of 0.4–0.5.

### PHB bead isolation and analysis

**Fig. 1:**
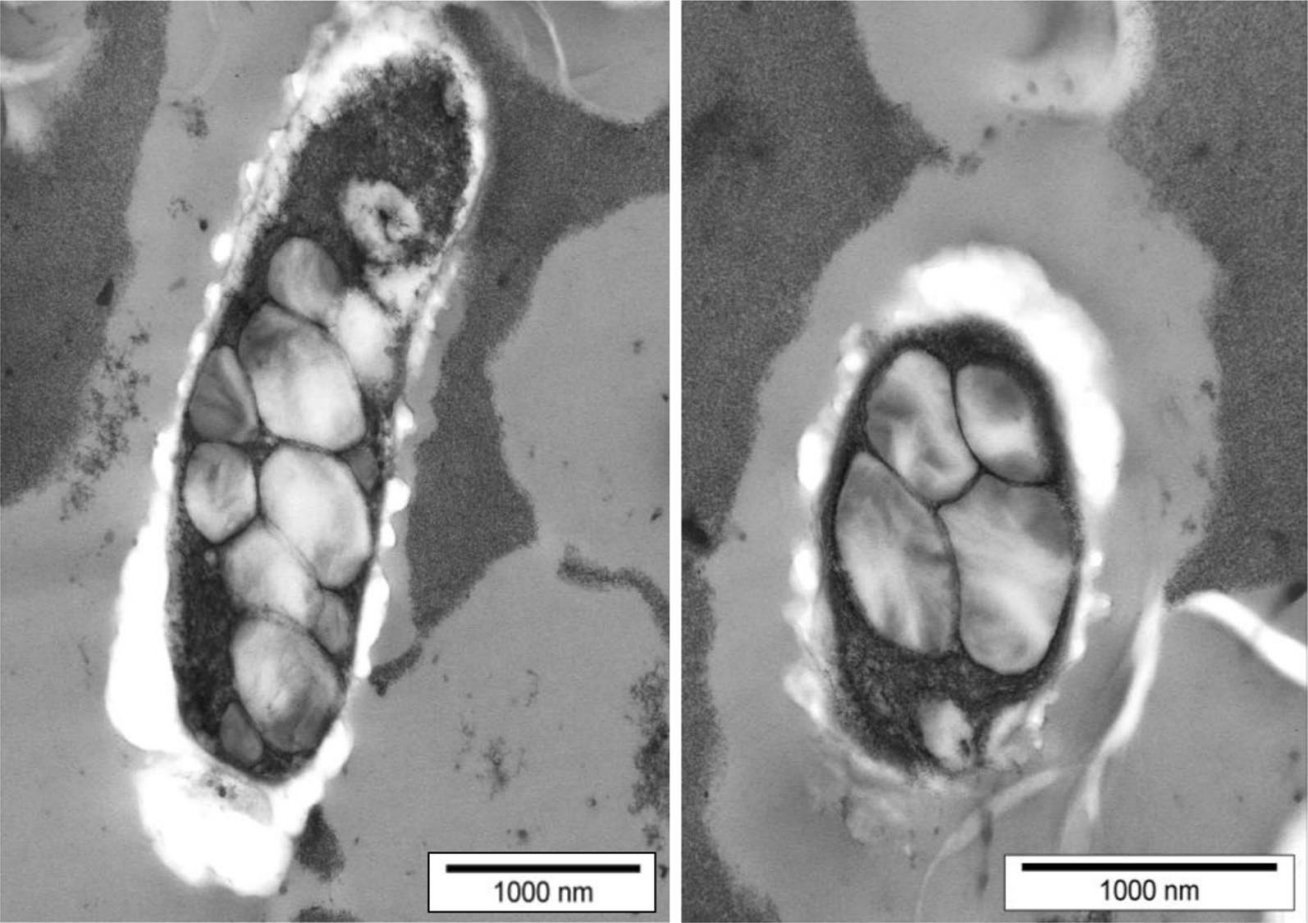
Transmission electron microscopic analysis of *B. megaterium* PHA05 cells containing recombinantly produced PHB beads. The ZZ-domain displaying beads were produced from plasmid pPT7-ZZCAB

**Fig. 2:**
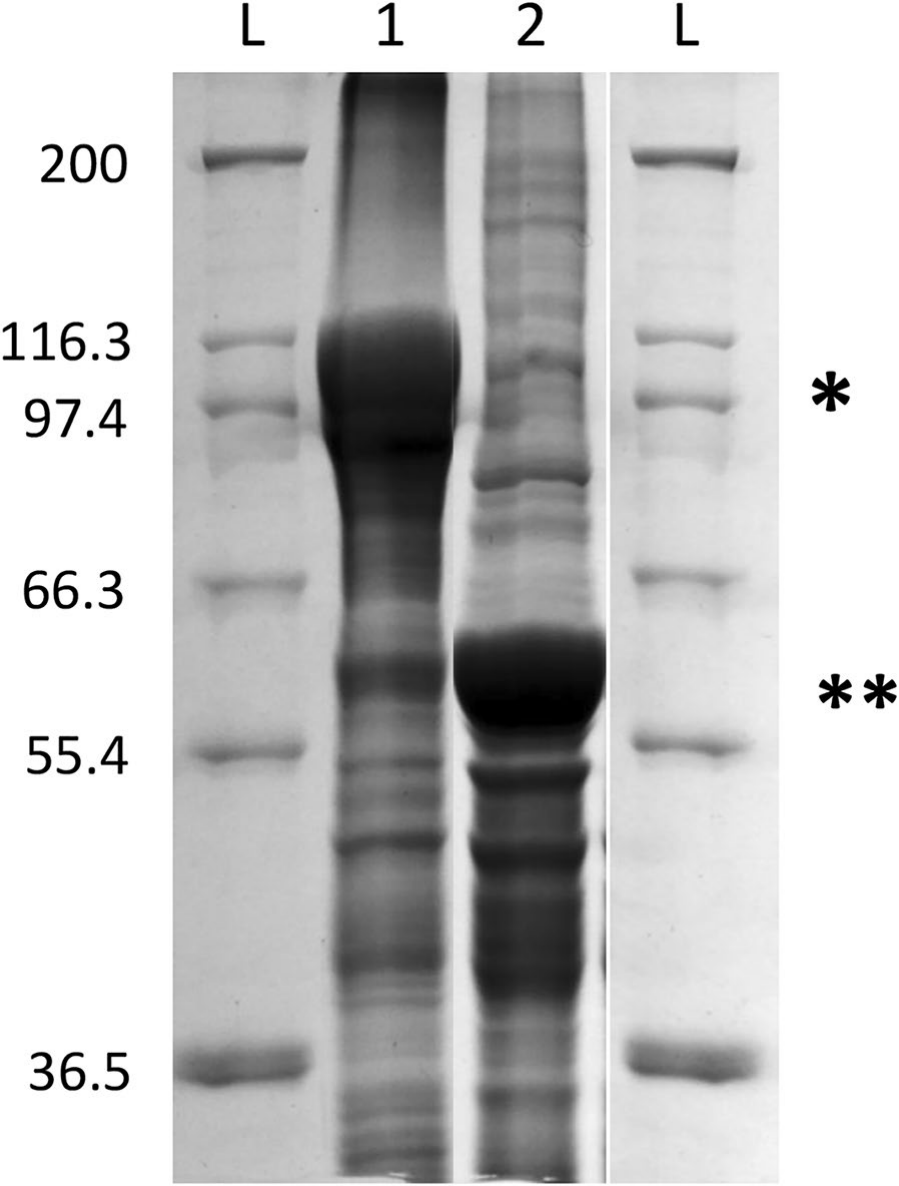
SDS-PAGE of ZZ-displaying and wild type beads recombinantly produced in *B. megaterium* PHA05 under control of the T7 promoter. Lane 1, ZZ-displaying beads, ZZ/ZZ-PhaC-ZZ protein at one asterisk (expected molecular weight 118 kDa); lane 2, wild type beads, PhaC protein at two asterisks (expected molecular weight 118 kDa). Equal amounts of protein as determined by Bradford were loaded in each lane. L, Mark 12™ protein standard (Thermo Fisher Scientific). The identity of the ZZ/ZZ-PhaC-ZZ fusion protein was confirmed by MALDI-TOF(MS)

**Fig. 3:**
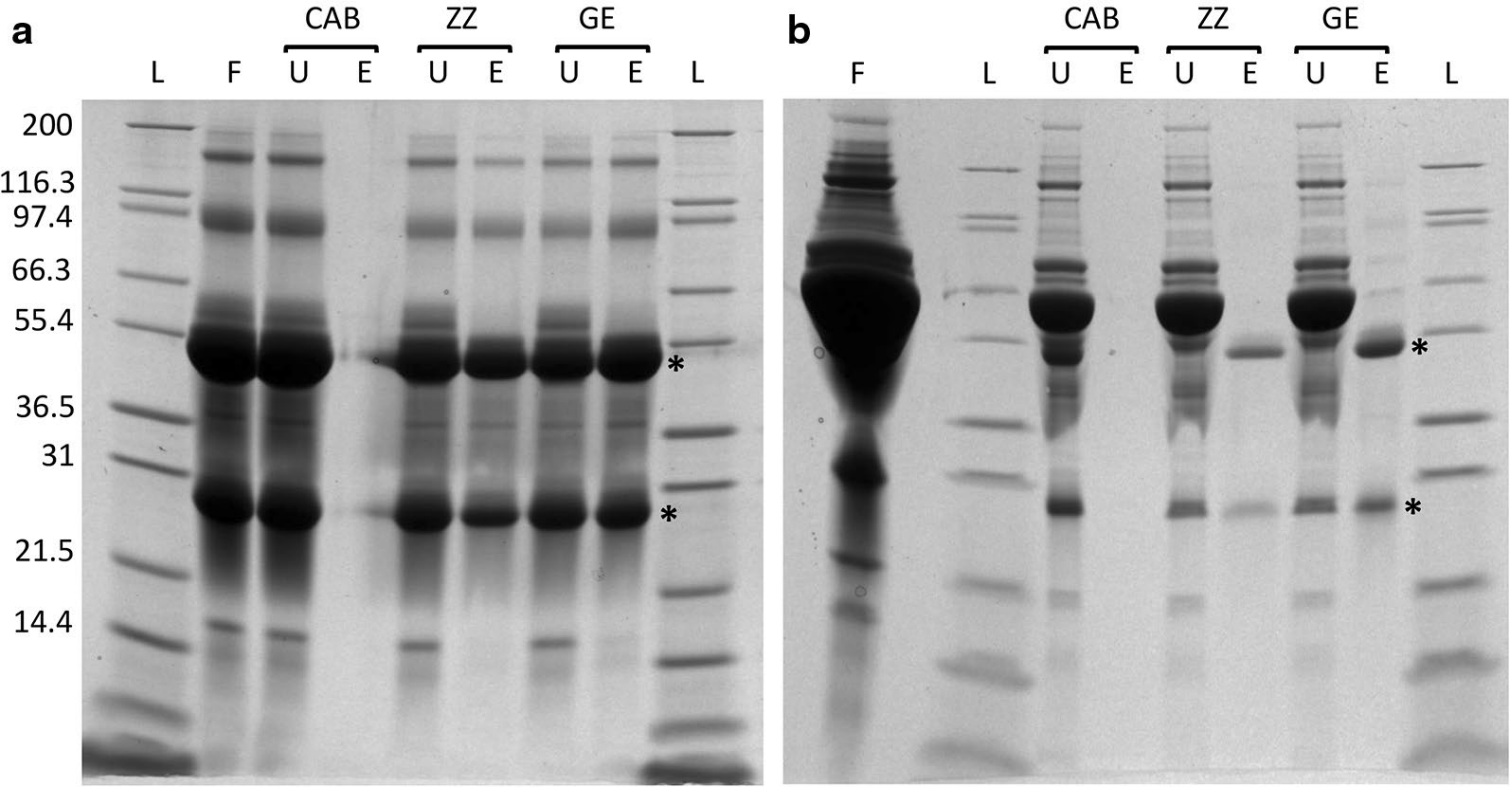
IgG binding assays using **a** purified IgG and **b** human serum. F, feed [IgG (**a**) or human serum (**b**)]; *U* unbound, *E* elution, *CAB* control beads not displaying ZZ domain, *ZZ* ZZ-domain displaying beads produced in this study, *GE* commercially available GE protein A beads; L, Mark 12™ protein standard (Thermo Fisher Scientific). Beads were incubated with the respective feed material, washed repeatedly and IgG eluted with glycine pH 2.7. Asterisks indicate the IgG light and heavy chains

PHB yields of cultures producing the ZZ-domain PhaC fusion were routinely quantified by GC/MS. The PHB content was typically in the range of 16–21% of cellular dry weight (CDW), which was similar to PHB yields obtained for PHA05 producing just wild type PhaC. Cells producing ZZ-domain displaying beads were also assessed by TEM (Fig. 1). PHB beads were then isolated from disrupted cells by density gradient centrifugation. Up to 2.3 g of beads (wet weight) were obtained from a biomass of 25–30 g cells (wet weight) produced per litre of culture. Beads were analysed by SDS-PAGE to assess the amount of fusion protein present at the bead surface (Fig. 2). Figure 2 shows that both PhaC and ZZ/ZZPhaC-ZZ were strongly overproduced at the bead surface (theoretical molecular weights 65 and 118 kDa, respectively). The identity of ZZ/ZZPhaC-ZZ was confirmed by MALDI-TOF(MS). Beads were further subjected to IgG binding assays, first testing binding and low pH elution using purified IgG and then by purifying IgG from human serum (Fig. 3). Using purified IgG, the beads showed a binding capacity of 40–50 mg IgG/g wet beads, compared to 60 mg IgG binding to commercial protein A beads. The ZZ-domain displaying PHB beads were also shown to successfully purify IgG from human serum as shown in Fig. 3. No IgG was eluted from control beads not displaying the ZZ-domains.

### Generation of a sporulation negative strain

**Fig. 4:**
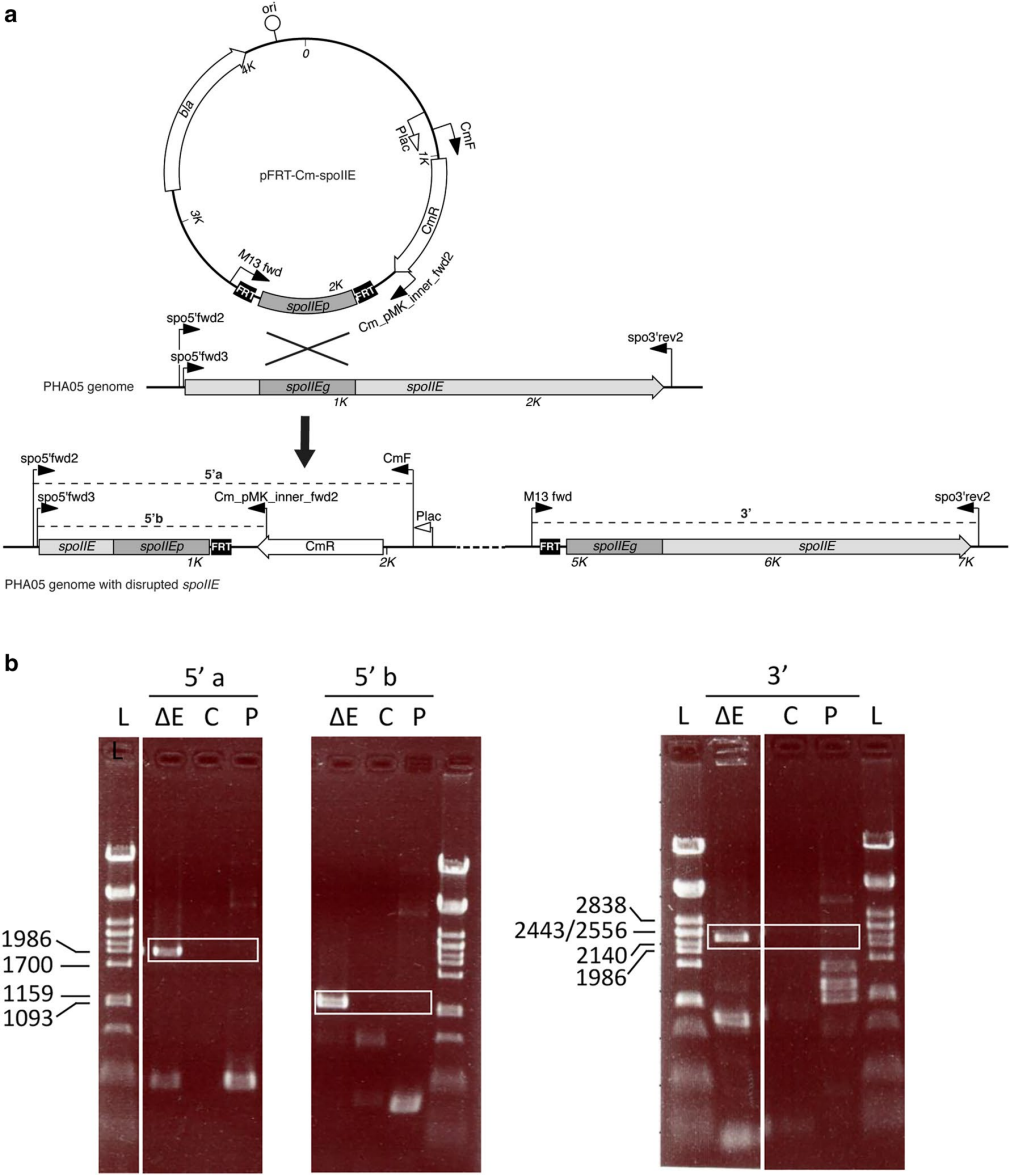
**a** Overview of *spoIIE* disruption by single crossover (Campbell-like integration) and **b** confirmation of *spoIIE* disruption by PCR amplification. **a** The constructed plasmid unable to replicate in the host organism is depicted. It contains a section of the target gene (between FRT sites) plus a selectable marker (Cm^R^). Homologous recombination occurs between the gene fragment on the plasmid and the identical gene segment on the chromosome followed by genome integration of the entire plasmid. The target gene is ‘insertionally inactivated’, i.e. disrupted by splitting it into a truncated forward and rear end (with the homologous gene sequence present twice in the altered genome). **b** Amplification across each junction was done using one primer binding in the genome and one primer binding within the integrated plasmid-derived fragment. 5′ a, amplification across 5′ junction using primers spo5′fwd2 and CmF (1.9 kb product); 5′ b, amplification across 5′ junction using primers spo5′fwd3 and Cm_pMK_inner_fwd2 (1.2 kb product); 3′, amplification across 3′ junction using primers M13 fwd and spo3′rev2 (2.3 kb product); ΔE, *spoIIE* knockout strain; C, *B. megaterium* PHA05 wild type control; P, plasmid control (pFRT-Cm-spoIIE). For supplemental confirmation, the PCR product obtained with primers spo5′-fwd2 and CmF was sequenced. L, ladder λ PstI (PstI digested λ DNA)

Since *B. megaterium* is capable of sporulation and since spore stains of harvested biomass from PHB bead production runs indicated the presence of some spores (Additional file 1: Figure S3), it was conceived to generate a sporulation-negative derivative of PHA05. Looking at early sporulation genes, one could either attempt to knock out *spo0A*, the master regulator for entry into spore formation, or a *spoII* gene [44–46]. SpoIIE has a dual role relatively early during the sporulation process [35, 36]. It is required for the initial asymmetric cell division that determines the fate of the cell while also responsible for compartment-specific gene expression in the forespore [37, 38]. Hence *spoIIE* was considered as a good target for the inactivation of sporulation while retaining physiologically active and viable cells. Initially, a knockout by double crossover was attempted following the method used to delete the major extracellular protease of *B. megaterium* [47]. However, this approach was unsuccessful. The *spoIIE* gene was then inactivated using a single crossover technique (Campbell-like integration), following the method used by Chen et al. to generate a *phaZ1* knockout in *B. megaterium* (Fig. 4a) [48]. This method enables disruption of the target gene through integration of the entire plasmid into the genome. *B. megaterium* PHA05 was transformed with gene disruption plasmid pFRT-Cm-spoIIE and the resulting transformants screened for chloramphenicol resistance. The disruption plasmid is based on pUC18 and cannot replicate in *B. megaterium*; chloramphenicol resistance thus indicates genome integration. Chloramphenicol resistant colonies were further analysed by PCR and sporulation assays. Disruption of the *spoIIE* gene was confirmed by PCR amplification of both ‘genome-plasmid insertion’ junctions (Fig. 4b). The PCR product obtained for the 5′ junction was sequenced and this further confirmed the successful disruption of *spoIIE*.

### Assessment of Δ*spoIIE* strain in regard to sporulation and PHB bead production

The newly generated Δ*spoIIE* strain was tested for its ability to form spores. It was shown to have a sporulation negative phenotype under the conditions tested (Table 1). In the sporulation assay, cells were first cultivated in liquid or on solid sporulation medium, then heat treated at 85 °C to kill all vegetative cells and finally dilution plated on LB to determine CFUs. To demonstrate that the sporulation medium does indeed promote sporulation, *B. megaterium* PHA05 was grown on this medium and used as a positive control. Exponentially growing PHA05 in LB was used as a negative control to show that the heat treatment kills non-sporulating cells. In all tests, all Δ*spoIIE* cells were killed by the heat treatment, indicating that by inactivating *spoIIE* a sporulation deficient derivative of *B. megaterium* PHA05 was successfully created.

**Table 1:** Sporulation assays

Strain	Time at 85 °C (min)^c^	From liquid culture		From solid medium		From solid medium	
		CFU/mL	Control (30 min 25 °C)	CFU/mL	Initial culture	CFU/mL	Initial culture
Positive control (WT spo)^a^	30	1.6 × 10^5^		3.0 × 10^6^		2.1 × 10^5^	
	45	2.2 × 10^5^	1.2 × 10^7^	7.1 × 10^5^	1.6 × 10^6^	2.3 × 10^5^	4.5 × 10^6^
	60	1.7 × 10^5^		9.6 × 10^4^		2.3 × 10^5^	
Δ*spoIIE*	30	0		0		0	
	45	0	2.4 × 10^6^	0	8.5 × 10^6^	0	1.6 × 10^6^
	60	0		0		0	
Negative control (WT exp)^b^	30	0		0		0	
	45	0	3.2 × 10^6^	0	8.7 × 10^5^	0	2.1 × 10^6^
	60	0		0		0	

^a^Positive control: *B. megaterium* PHA05 wild type, sporulates on/in sporulation medium

^b^Negative control: exponentially growing *B. megaterium* PHA05 wild type (in LB)

^c^85 °C had been experimentally determined to be the optimum temperature for this assay (all vegetative cells killed, spores survive)

Due to the presence of a chloramphenicol resistance gene within the *spoIIE* disruption site, the newly generated sporulation negative strain is currently incompatible with the T7 system which relies on the T7 RNA polymerase encoding plasmid pT7-RNAP that mediates resistance to chloramphenicol. Therefore, bead production in the new strain was assessed using the xylose inducible system based on pMM1522. While the initial plasmid constructs for the production of ZZ-domain displaying PHB beads were designed to result in an identical amino acid sequence to the *E. coli* system (for better comparability), these constructs still contain the original *S. aureus* signal sequence (SS). As this might have a more significant effect on the Gram-positive host, it was decided to remove the SS from pMM-ZZCAB and pT7-ZZCAB. An assessment of PHB bead production in *B. megaterium* PHA05 Δ*spoIIE* was carried out using pMM-ZZCAB(-SS). Both PHB content (approx. 5% of CDW for both) and IgG binding (33 mg/g beads (Δ*spoIIE*) versus 32 mg/g beads (PHA05)) were found to be comparable for the Δ*spoIIE* strain and its parent strain PHA05, indicating that the inactivation of *spoIIE* had no adverse effect on the suitability of this strain as a host for the recombinant production of engineered PHB beads.

## Discussion

In this study, we have explored the possible use of the Gram-positive endotoxin-free host *B. megaterium* for the production of functional intracellularly formed PHB beads. Here, we present proof-of-concept for the recombinant production of PHB in *B. megaterium*. The main focus was on producing functionalised beads such as PHB beads displaying the IgG binding ZZ-domain from *S. aureus*. Recombinant production was established in the PHB-negative *B. megaterium* strain PHA05 by introducing the genes *phaC*, *phaA* and *phaB,* which enable PHB biosynthesis from acetyl-CoA, under T7 promoter control on plasmid pPT7-CAB or pPT7-ZZCAB. Growing PHA05 with plasmids pT7-RNAP and pPT7-ZZCAB in glucose-supplemented A5 medium, up to 20% PHB of CDW was obtained. This is a significant improvement compared to engineered *Lactococcus lactis* which only accumulated up to 6% PHB of CDW [31]. As expected, the metabolic background of *B. megaterium*, a natural PHB producer, seems to be more suitable for PHB production. Higher PHB yields have been achieved in other recombinant organisms (e.g. *E. coli* or *Corynebacterium glutamicum*). In *C. glutamicum,* which originally reached PHB levels around 20%, apart from codon optimization, gene dosage made a crucial difference [49, 50]. In particular the expression levels of *phaA* and *phaB* relative to *phaC* were found to be critical. However, *C. glutamicum* was engineered with the aim of producing bulk PHB as a biodegradable plastic, whereas in the present study, the focus is on producing functionalised PHB beads. More important than the overall PHB content is thus a high-density display of the desired function (e.g. ZZ/ZZPhaC-ZZ) at the bead surface. The protein profile of purified beads (Fig. 2) showed strong overproduction of the ZZ-domain PhaC fusion protein. This was also reflected by an IgG binding capacity and purification power which resembled the performance of commercially available protein A beads (Fig. 3).

To exclude the possibility of sporulation and potential co-purification of spores with the beads, an improved *B. megaterium* bead production host was engineered by generating a sporulation-negative derivative of PHA05. Another sporulation-negative *B. megaterium* strain has previously been published [51]. However, in this strain the *spoIV* gene (stage IV = spore cortex synthesis) had been deleted, which still enables formation of pre-spores that still could co-purify with inclusions such as PHB beads. In addition, the existing Δ*spoIV* strain is not PHB-negative. In order to avoid pre-spore formation, we considered early stage sporulation genes such as *spo0A* or one of the *spoII* genes [44–46]. The *spo0A* gene encodes a master regulator that regulates other genes which in turn regulate post-exponential gene expression (e.g. prevent certain stationary phase functions from being expressed during exponential phase) [52–55]. Hence deleting *spo0A* could have unpredictable and undesired side-effects. Spo0A directly regulates several stage II sporulation genes such as the *spoIIA* and *spoIIG* operons (encoding sigma factors F and E) as well as *spoIIE* [44]. SpoIIE has been labelled the ‘cell fate determinant’ [56], a name that reflects its crucial role during the early stages of sporulation. It is a bifunctional protein involved first in asymmetric cell division and then in forespore-specific gene expression via sigma F activation [57–60]. When *spoIIE* was mutated in *Bacillus subtilis*, sporulation did not occur. Asymmetric cell division (due to FtsZ) still happened but in a lower percentage of cells [58]. In a *Clostridium acetobutylicum spoIIE* disruption strain, sporulation was even blocked prior to asymmetric division and no morphogenetic changes were observed [61]. Interestingly, Bi et al. also had difficulties inactivating the *C. acetobutylicum spoIIE* gene by double crossover and only achieved the disruption by single crossover [61]. For this study, it was irrelevant whether asymmetric cell-division still occurred in the mutant or not. Thus, the *spoIIE* gene was considered as a suitable target for inactivation. Here, the *spoIIE* gene was disrupted in the PHB-negative strain *B. megaterium* PHA05 (Fig. 4), abolishing the ability to sporulate as shown by 100% killing after heat treatment in the sporulation assays (Table 1). Unfortunately, attempts to remove the chloramphenicol resistance gene from the knockout via the flanking FRT sites have so far been unsuccessful. Therefore, the Δ*spoIIE* strain is currently incompatible with the T7 expression system as pT7RNAP contains a Cm^R^ cassette. The strain’s ability to sustain the production of functionalized (ZZ-domain displaying) PHB beads was thus preliminarily assessed using the xylose-inducible system based on pMM1522 [pMM-ZZCAB(-SS)]. Both PHB content and IgG binding performance were comparable for Δ*spoIIE* and its parent strain PHA05, suggesting that the *spoIIE* gene disruption had no adverse effect on growth or PHB production.

In addition to restoring compatibility of the T7 system with the *spoIIE* disruption strain, possible approaches to enhance productivity and performance could include gene dosage and additional Z-domains. In *C. glutamicum*, increasing the copy number of *phaA* and *phaB* (by supplying an additional copy on a second plasmid in addition to the plasmid bearing *phaC*, *phaA* and *phaB*) significantly increased PHB yield [50]. In *E. coli*, on the other hand, high PHB yields have routinely been obtained by expressing *phaC* under T7 promoter control and *phaA* and *phaB* under *lac* promoter control [16, 62]. Recently, Hooks et al. identified regions within PhaC that tolerated insertions, e.g. of ZZ-domains [63]. Some of the newly created ZZ-domain PhaC fusions exhibited improved IgG binding. These strategies could be explored to increase PHB bead production and the density of ZZ-domains displayed at the bead surface in order to improve cost-effectiveness of production as well as PHB bead performance when using *B. megaterium* PHA05 ∆*spoIIE*.

## Conclusions

This study demonstrates the successful genetic engineering of *B. megaterium* as a host for the production of functionalized PHB-based biobeads. ZZ-domain displaying PHB beads suitable for purification of IgG from human serum were produced. Functional PHB bead production was mediated by T7 promoter-dependent expression of relevant genes in the PHB-negative strain PHA05. As sporulation occurred during PHB bead production a sporulation negative derivative of PHA05 was generated by disruption of the *spoIIE* gene. The sporulation- and PHB-negative *B. megaterium* strain generated in this study, represents an industrial host suitable for the production of intracellular products such as functional PHB beads or inclusion bodies.

## Methods

### Plasmids, DNA constructs and oligonucleotides

All plasmids are listed in Table 2. All oligonucleotides are listed in Additional file 1: Table S5. To make the PHB-production plasmids, genes were synthesized with their sequence optimized for Gram-positive codon usage. The ZZ/ZZC-ZZ encoding sequences were designed to translate into a protein with identical amino acid sequence compared to the *E. coli* produced protein [16]. *phaC*, *phaA*, *phaB*, the N-terminal Z domains and the C-terminal Z domains were all synthesized individually by GenScript and provided in plasmid pUC57. Restriction sites for cloning were included in the gene synthesis. To generate plasmid pMM-AB, pUC57-*phaA* and pUC57-*phaB* were digested BglII-BamHI and BamHI-KpnI, respectively, and the genes *phaA* and *phaB* ligated into BglII-KpnI digested vector pMM1522 (Table 2) [34]. pUC57-*phaC* and pMM-AB were then digested BsrGI-BglII and *phaC* ligated into pMM-AB to result in plasmid pMM-CAB. To generate plasmid pMM-ZZCAB, the N-terminal Z domains were excised from pUC57 with BsrGI and PciI and ligated into BsrGI-NcoI digested pUC57-*phaC*. The resulting plasmid was digested BsrGI-Bsu361, pUC57 containing the C-terminal Z domains was digested Bsu361-BglII, and the resulting two fragments ZZ/ZZ-phaC(5′) and phaC(3,)-ZZ were ligated into BsrGI-BglII digested plasmid pMM-CAB, thereby replacing *phaC* with ZZ/ZZ*phaC*-ZZ. pPT7-AB and pPT7-CAB were generated by cloning the respective fragments AB and CAB from pMM-AB and pMM-CAB into pPT7 (BglII-KpnI and BsrGI-KpnI, respectively) [33]. pPT7-ZZCAB was constructed in the same way as described above for pMM-ZZCAB (replacing *phaC* with ZZ/ZZ*phaC*-ZZ). p1623-ZZCAB was generated by cloning the entire ZZ/ZZC-ZZ-AB fragment of pMM-ZZCAB BsrGI-KpnI into p1623hp [35].

**Table 2:** Strains and plasmids used in this study

Strain/plasmid name	Genotype/description	Source/references
Strain		
*E. coli* XL1BLue	*recA1 endA1 gyrA96 thi*-*1 hsdR17 supE44 relA1 lac [F′ proAB lacI* ^*q*^ *lacZ ΔM15 Tn10 (Tet* ^*R*^ *)]*	Stratagene
*B. megaterium* PHA05	PHA-negative mutant of 11561 (ATCC); Δ(*phaP*-*phaC*); Em^r^	[41]
*B. megaterium* PHA05 ΔspoIIE	Sporulation-negative mutant of PHA05; Δ(*phaP*-*phaC*) Δ*spoIIE*; Em^R^ Cm^R^	This study
Plasmid		
pUC57-*phaA*	Codon optimized *phaA* in pUC57, Amp^R^	GenScript
pUC57-*phaB*	Codon optimized *phaB* in pUC57, Amp^R^	GenScript
pUC57-*phaC*	Codon optimized *phaC* in pUC57, Amp^R^	GenScript
pUC57-ZZ/ZZ	Codon optimized N-terminal Z domains in pUC57, Amp^R^	GenScript
pUC57-ZZ	Codon optimized C-terminal Z domains in pUC57, Amp^R^	GenScript
pMM1522	Shuttle vector for cloning in *E. coli* (Amp^R^) and expression under xylose control in *B. megaterium* (Tet^R^)	[34]
pMM-AB	Codon optimized *phaA* and *phaB* cloned BglII-BamHI and BamHI-KpnI, respectively, into pMM1522	This study
pMM-CAB	Codon optimized *phaC* cloned BsrGI-BglII into pMM-AB	This study
pMM-ZZCAB	ZZ/ZZC-ZZ cloned BsrGI-BglII into pMM-CAB, replacing *phaC*	This study
pPT7	Shuttle vector for cloning in *E. coli* (Amp^R^) and expression in *B. megaterium* (Tet^R^). T7 promoter and terminator inserted to give P_T7_-MCS-Stop-T_T7_	[33]
pPT7-AB	*phaAB* fragment from pMM-AB cloned BglII-KpnI into pPT7	This study
pPT7-CAB	*phaCAB* fragment from pMM-CAB cloned BsrGI-KpnI into pPT7	This study
pPT7-ZZCAB	ZZ/ZZC-ZZ cloned BsrGI-BglII into pPT7-CAB, replacing *phaC*	This study
pT7RNAP	Helper plasmid bearing T7 RNA polymerase under control of xylA promoter (Cm^R^)	[33]
p1623hp (=p3STOP1623hp)	Shuttle vector for cloning in *E. coli* (Amp^R^) and expression under xylose control in *B. megaterium* (Tet^R^)	[35]
p1623-ZZCAB	ZZ/ZZCZZ-AB fragment from pMM-ZZCAB cloned BsrGI-KpnI into p1623hp	This study
pPT7-ZZCAB(-SS)	N-terminal ZZ/ZZ region PCR amplified (without SS) from pPT7-ZZCAB and re-inserted into pPT7-CAB; C-terminal Z domains sucloned Bsu361-KpnI from pPT7-ZZCAB	This study
pMM1522PacI	G-block fragment ‘SalI-part of xylR-PxylA-PacI-SpeI’ inserted SalI-SpeI into pMM1522	This study
pMM-ZZCAB(-SS)	ZZ/ZZC-ZZ-AB fragment subcloned PacI-KpnI from pPT7-ZCAB(-SS) to give pMM-ZCAB(-SS)	This study
pUC18	*E. coli* cloning vector, Amp^R^. No origin of replication for *B. megaterium*	Thermo scientific
pMK-RQ-Cm	Gene synthesis of chloramphenicol resistance gene, flanked by FRT sites (FRT-Cm^R^-FRT) in pMK-RQ, Kan^R^	GeneArt
pUC18-FRT = pFRT	FRT sites (amplified from pMK-RQ-Cm (synthesis of FRT-Cm^R^-FRT)) cloned HindIII-FRT-BamHI-FRT-KpnI into pUC18	This study
pFRT-Cm	Cm^R^ gene PCR amplified from pMK-RQ-Cm with primers Cm-Fpsc and Cm-Rpsc (introducing SacI sites) and cloned SacI into pFRT	This study
pFRT-Cm-spoIIE	500 nt fragment of *B. megaterium spoIIE* gene PCR amplified with primers SpoF-psc (NheI) and SpoR-psc (ApaI) and cloned NheI-ApaI into pFRT-Cm	This study

To remove the sequence for the N-terminal signal peptide of the Z domains, a PacI restriction site was inserted into plasmid pMM1522 upstream of the original RBS: A 500 bp fragment comprising SalI—part of xylR–PxylA–PacI–SpeI was designed and ordered as a G-block from IDT. This region was then cloned SalI-SpeI into pMM1522, generating pMM1522PacI. The entire ZZ/ZZC-ZZ-AB fragment of pPT7-ZZCAB(-SS) was then subcloned PacI-KpnI into pMM1522PacI to give pMM-ZZCAB(-SS). To generate pPT7-ZCAB(-SS), the N-terminal ZZ/ZZ containing region was amplified from the original pPT7-ZZCAB with primers ZZ-fwd-noSS and ZZ-rev-BspEI and re-inserted into pPT7-CAB (PacI, BspEI). The C-terminal Z domains were added by subcloning the C-terminal region of ZZ/ZZphaC-ZZ from pPT7-ZZCAB Bsu361-BglII into the above plasmid.

To generate the single crossover *spoIIE* disruption vector starting from pUC18, a DNA fragment comprising FRT-Cm^R^-FRT was synthesized by GeneArt (pMK-RQ-Cm). The FRT sites were PCR amplified with primers that inserted restriction sites (FRT-1Fpsc (HindIII) and FRT-1Rpsc (BamHI) or FRT-2Fpsc (BamHI) and FRT-2Rpsc (KpnI)) and then cloned into pUC18 HindIII-BamHI and BamHI-KpnI, respectively, resulting in pFRT. The Cm^R^ gene was then also PCR amplified from pMK-RQ-Cm (with primers CmF-psc and CmR-psc (introducing SacI sites)) and cloned SacI into pFRT, resulting in pFRT-Cm. Finally, a 500-nucleotide-fragment of the *B. megaterium spoIIE* gene was PCR amplified (with primers SpoF-psc (NheI) and SpoR-psc (ApaI)) and cloned NheI-ApaI into pFRT-Cm, generating the spoIIE disruption vector pFRT-Cm-spoIIE.

### Bacterial strains and growth conditions

All strains are listed in Table 2. *E. coli* was routinely grown in LB medium at 37 °C. *B. megaterium* was grown in LB at 37 °C or—for PHB production—A5 medium supplemented with 2% (w/v) glucose. For PHB production, *B. megaterium* bearing the relevant plasmid(s) was initially cultivated at 37 °C, induced by the addition of xylose to a final concentration of 0.5% (w/v) or 1% (w/v) at OD_600_ of 0.4–0.5, and then cultivated at 25 °C for another 24 h.

*A5 medium* (modified from [39, 43]): 2 g/L (NH_4_)_2_SO_4_, 3.52 g/L KH_2_PO_4_, 5.683 g/L Na_2_HPO_4_, 0.3 g/L Mg SO_4_·7H_2_O, 1 g/L yeast extract, 1 mL/L trace element stock solution [added after autoclaving), 100 mL/L glucose (20% (w/v)] (added after autoclaving). Trace element stock solution (filter sterilized): 40 g/L MnCl_2_·4H_2_O, 53 g/L CaCl_2_·2H_2_O, 2 g/L (NH_4_)_6_MO_7_O_24_·4H_2_O, 2 g/L CoCl_2_·6H_2_O, 2.5 g/L FeSO_4_·7H_2_O.

*Antibiotics* Ampicillin (75 μg/mL for *E. coli*), chloramphenicol (4.5 μg/mL) and tetracycline (10 μg/mL).

*Sporulation medium* (modified from [64]): 3 g/L beef extract, 5 g/L peptone, 1 g/L KCl, 10 mL/L of 2% MgSO_4_ × 7H_2_O, pH 7–7.5; after autoclaving, add: 1 mL of 1 M CaCl_2_ × 2H_2_O, 100 μL of 0.1 M MnCl_2_ × 4H_2_O, 100 μL of 10 mM FeSO_4_ × 7H_2_O; used as liquid medium and agar plates. Strains were grown in liquid or on solid sporulation medium at 37 °C for one or 2 days. A colony or an aliquot of a cell pellet was resuspended in 50 μL of sterile water or Tris buffer, heated to 85 °C for 30, 45 or 60 min and spotted on LB plates.

### Transformation procedures

*Escherichia coli* was transformed by heat shock of chemically competent cells as described previously [65]. *B. megaterium* was transformed by protoplast transformation following the protocols published by Barg et al. and Biedendieck et al. [36, 66].

### Isolation of polymer beads

PHB beads were isolated as described previously with modifications to allow for the new host and new plasmid system [31, 62]. *B. megaterium* PHA05 containing plasmids pT7-RNAP (Cm^R^) and pPT7-ZZZZCZZ-AB (Tet^R^) (or one of the other plasmid systems, with the respective antibiotics) was cultivated in A5 medium with 10 μg/mL tetracycline and 4.5 μg/mL chloramphenicol at 37 °C. At OD_600_ of 0.4–0.5, protein production was induced by addition of xylose (0.5 or 1% final concentration) and the cultures were shifted to 25 °C. After 24 h of growth cells were harvested by centrifugation. After resuspension in 50 mM phosphate buffer (pH 7.4) cells were disrupted by lysozyme treatment (30 min at 37 °C) and cell disruptor passage (2 passages at 30 kpsi). Following centrifugation and resuspension of the pellet (crude beads) in phosphate buffer, the material was loaded onto an 88 and 44% (vol/vol) glycerol gradient and subjected to ultracentrifugation at 100.000×*g* for 2 h. Beads were retrieved from the interface between the 88 and 44% glycerol layers.

### Staining techniques

In order to visualize PHB inclusions inside *B. megaterium* cells for a quick assessment of PHB production, cells were stained with the lipophilic dye Nile Red. 100 µL of culture were pelleted by centrifugation, the pellet washed with and resuspended in 1 mL of potassium phosphate buffer (pH 7.4) and 10 µL of a Nile Red stock solution (250 µg/mL in DMSO) added. After incubation in the dark at room temperature for 5–15 min, cells were washed to remove exess Nile Red, mounted on microscope slides and examined under the fluorescene microscope (Olympus BX51 Microscope).

Bacterial endospores were stained with malachite green. Briefly, a resuspended culture aliquot was smeared on a microscope slide and briefly left to air dry. The smear was then flooded with malachite green and the dye heat fixed. Excess dye was washed off with water, and vegetative cells were counterstained with aqueous safranin for 30 s. Excess dye was again washed off, slides carefully blotted dry and observed under the oil immersion (Zeiss Axiophot Microscope). All microscopy was done at the Manawatu Microscopy and Imaging Centre (Palmerston North, New Zealand).

### Transmission electron microscopy (TEM) analysis

To confirm PHB bead formation inside recombinant *B. megaterium* and to visually assess the beads, TEM analysis was performed at the Manawatu Microscopy and Imaging Centre (Palmerston North, New Zealand). After 24 h of growth, cells were harvested, washed with 50 mM potassium phosphate buffer (pH 7.4), and sediments were prepared for TEM as described previously [62].

### PHA quantification by gas chromatography/mass spectrometry (GC/MS)

In vivo activity of the polyester synthase was obtained by analysing the polyester content of the respective bacterial cells. PHA content of the lyophilized cells was quantified using gas chromatography-mass spectrometry (GC/MS) after conversion of the PHA into 3-hydroxymethylester by acid-catalyzed methanolysis [67].

### Protein analysis

Protein samples (including bead-bound proteins) were routinely analysed by SDS-PAGE (sodium dodecyl sulphate-polyacrylamide electrophoresis) as described elsewhere [68]. The gels were stained with Coomassie brilliant blue G250. Protein concentrations were determined using the Bio-Rad Protein Assay. Dominant bands were excised from the gel and identified by matrix-assisted laser desorption ionization-time of flight (mass spectrometry) [MALDI-TOF(MS)] as described previously [15].

### IgG binding assays

The IgG binding performance (binding capacity and purification power) of purified ZZ-displaying beads was assessed and quantified by two types of assays as previously described: binding and elution of purified IgG and purification of IgG from human serum [14, 31]. Briefly, a defined amount of beads was incubated with the feed material (IgG or serum), subjected to a series of washes and bound IgG eluted with glycine pH2.7. Elution fractions were assessed by SDS-PAGE analysis.

## Additional file

**Additional file 1.** Additional tables and figures.

## References

[1] Luo X, Bathgate RA, Liu YL, Shao XX, Wade JD, Guo ZY (2009). Recombinant expression of an insulin-like peptide 3 (INSL3) precursor and its enzymatic conversion to mature human INSL3. FEBS J.

[2] Zhou Y, Ma X, Hou Z, Xue X, Meng J, Li M, Jia M, Luo X (2012). High cell density cultivation of recombinant *Escherichia coli* for prodrug of recombinant human GLPs production. Protein Expr Purif.

[3] Rehm FB, Chen S, Rehm BHA (2016). Enzyme engineering for in situ immobilization. Molecules.

[4] Rehm FBH, Chen S, Rehm BHA. Bioengineering toward direct production of immobilized enzymes: a paradigm shift in biocatalyst design. Bioengineered. 2017;1–6. 10.1080/21655979.2017.1325040. 10.1080/21655979.2017.1325040 PMC5972917 28463573

[5] Jahns AC, Maspolim Y, Chen S, Guthrie JM, Blackwell LF, Rehm BHA (2013). In vivo self-assembly of fluorescent protein microparticles displaying specific binding domains. Bioconjug Chem.

[6] Venning-Slater M, Hooks DO, Rehm BHA (2014). In vivo self-assembly of stable green fluorescent protein fusion particles and their uses in enzyme immobilization. Appl Environ Microbiol.

[7] Rehm BHA (2017). Bioengineering towards self-assembly of particulate vaccines. Curr Opin Biotechnol.

[8] Peters V, Rehm BHA (2006). *In vivo* enzyme immobilization by use of engineered polyhydroxyalkanoate synthase. Appl Environ Microbiol.

[9] Rehm BHA (2003). Polyester synthases: natural catalysts for plastics. Biochem J.

[10] Hay ID, Du J, Burr N, Rehm BHA (2015). Bioengineering of bacteria to assemble custom-made polyester affinity resins. Appl Environ Microbiol.

[11] Jahns AC, Rehm BHA (2015). Immobilization of active lipase B from *Candida antarctica* on the surface of polyhydroxyalkanoate inclusions. Biotech Lett.

[12] Chen SX, Parlane NA, Lee J, Wedlock DN, Buddle BM, Rehm BHA (2014). New skin test for detection of bovine tuberculosis on the basis of antigen-displaying polyester inclusions produced by recombinant *Escherichia coli*. Appl Environ Microbiol.

[13] Grage K, Peters V, Rehm BHA (2011). Recombinant protein production by in vivo polymer inclusion display. Appl Environ Microbiol.

[14] Lewis JG, Rehm BHA (2009). ZZ polyester beads: an efficient and simple method for purifying IgG from mouse hybridoma supernatants. J Immunol Methods.

[15] Jahns AC, Haverkamp RG, Rehm BHA (2008). Multifunctional inorganic-binding beads self-assembled inside engineered bacteria. Bioconjug Chem.

[16] Brockelbank JA, Peters V, Rehm BHA (2006). Recombinant *Escherichia coli* strain produces a ZZ domain displaying biopolyester granules suitable for immunoglobulin G purification. Appl Environ Microbiol.

[17] Lee JW, Parlane NA, Rehm BHA, Buddle BM, Heiser A (2017). Engineering mycobacteria for the production of self-assembling biopolyesters displaying mycobacterial antigens for use as a tuberculosis vaccine. Appl Environ Microbiol.

[18] Lee JW, Parlane NA, Wedlock DN, Rehm BHA (2017). Bioengineering a bacterial pathogen to assemble its own particulate vaccine capable of inducing cellular immunity. Sci Rep.

[19] Rehm BHA (2007). Biogenesis of microbial polyhydroxyalkanoate granules: a platform technology for the production of tailor-made bioparticles. Curr Issues Mol Biol.

[20] Draper JL, Rehm BHA (2012). Engineering bacteria to manufacture functionalized polyester beads. Bioengineered.

[21] Grage K, Jahns AC, Parlane N, Palanisamy R, Rasiah IA, Atwood JA, Rehm BHA (2009). Bacterial polyhydroxyalkanoate granules: biogenesis, structure, and potential use as nano-/micro-beads in biotechnological and biomedical applications. Biomacromol.

[22] Rehm FBH, Grage K, Rehm BHA, Lee SY (2016). Applications of microbial biopolymers in display technology. Consequences of microbial interactions with hydrocarbons, oils, and lipids: production of fuels and chemicals.

[23] Rehm BHA: Process for the production of biodegradeable, functionalised polymer particles, and use thereof as pharmaceutical supports. US Patent Application 14/143,725. 2013.

[24] Rehm BHA: Method for producing biodegradable, functionalised polymer particles, and use of the same as medicament carriers. WO 2004020623 A3. 2004.

[25] Valappil SP, Boccaccini AR, Bucke C, Roy I (2007). Polyhydroxyalkanoates in Gram-positive bacteria: insights from the genera *Bacillus* and *Streptomyces*. Antonie Van Leeuwenhoek.

[26] Valappil SP, Misra SK, Boccaccini AR, Keshavarz T, Bucke C, Roy I (2007). Large-scale production and efficient recovery of PHB with desirable material properties, from the newly characterised *Bacillus cereus* SPV. J Biotechnol.

[27] Valappil SP, Rai R, Bucke C, Roy I (2008). Polyhydroxyalkanoate biosynthesis in *Bacillus cereus* SPV under varied limiting conditions and an insight into the biosynthetic genes involved. J Appl Microbiol.

[28] Dhangdhariya JH, Dubey S, Trivedi HB, Pancha I, Bhatt JK, Dave BP, Mishra S (2015). Polyhydroxyalkanoate from marine *Bacillus megaterium* using CSMCRI’s Dry Sea Mix as a novel growth medium. Int J Biol Macromol.

[29] Tsuge T, Hyakutake M, Mizuno K (2015). Class IV polyhydroxyalkanoate (PHA) synthases and PHA-producing *Bacillus*. Appl Microbiol Biotechnol.

[30] Xiao ZJ, Zhang Y, Xi LJ, Huo FF, Zhao JY, Li J (2015). Thermophilic production of polyhydroxyalkanoates by a novel *Aneurinibacillus* strain isolated from Gudao oilfield, China. J Basic Microb.

[31] Mifune J, Grage K, Rehm BHA (2009). Production of functionalized biopolyester granules by recombinant *Lactococcus lactis*. Appl Environ Microbiol.

[32] Lemoigne M (1926). Produits de déshydratation et de polymérisation de l’acide oxybutyrique. Cr Soc Biol.

[33] Gamer M, Frode D, Biedendieck R, Stammen S, Jahn D (2009). A T7 RNA polymerase-dependent gene expression system for *Bacillus megaterium*. Appl Microbiol Biotechnol.

[34] Malten M, Biedendieck R, Gamer M, Drews AC, Stammen S, Buchholz K, Dijkhuizen L, Jahn D (2006). A *Bacillus megaterium* plasmid system for the production, export, and one-step purification of affinity-tagged heterologous levansucrase from growth medium. Appl Environ Microbiol.

[35] Stammen S, Muller BK, Korneli C, Biedendieck R, Gamer M, Franco-Lara E, Jahn D (2010). High-yield intra- and extracellular protein production using *Bacillus megaterium*. Appl Environ Microbiol.

[36] Biedendieck R, Borgmeier C, Bunk B, Stammen S, Scherling C, Meinhardt F, Wittmann C, Jahn D (2011). Systems Biology of recombinant protein production using *Bacillus megaterium*. Methods Enzymol.

[37] Korneli C, Biedendieck R, David F, Jahn D, Wittmann C (2013). High yield production of extracellular recombinant levansucrase by *Bacillus megaterium*. Appl Microbiol Biotechnol.

[38] Korneli C, David F, Biedendieck R, Jahn D, Wittmann C (2013). Getting the big beast to work: systems biotechnology of *Bacillus megaterium* for novel high-value proteins. J Biotechnol.

[39] Malten M, Hollmann R, Deckwer WD, Jahn D (2005). Production and secretion of recombinant *Leuconostoc mesenteroides* dextransucrase DsrS in *Bacillus megaterium*. Biotechnol Bioeng.

[40] Vary PS, Biedendieck R, Fuerch T, Meinhardt F, Rohde M, Deckwer WD, Jahn D (2007). *Bacillus megaterium:* from simple soil bacterium to industrial protein production host. Appl Microbiol Biotechnol.

[41] McCool GJ, Cannon MC (2001). PhaC and PhaR are required for polyhydroxyalkanoic acid synthase activity in *Bacillus megaterium*. J Bacteriol.

[42] Rehm BH (2010). Bacterial polymers: biosynthesis, modifications and applications. Nat Rev Microbiol.

[43] Hollmann R, Deckwer WD (2004). Pyruvate formation and suppression in recombinant *Bacillus megaterium* cultivation. J Biotechnol.

[44] Hilbert DW, Piggot PJ (2004). Compartmentalization of gene expression during *Bacillus subtilis* spore formation. Microbiol Mol Biol Rev.

[45] Piggot PJ, Hilbert DW (2004). Sporulation of *Bacillus subtilis*. Curr Opin Microbiol.

[46] Yudkin MD, Clarkson J (2005). Differential gene expression in genetically identical sister cells: the initiation of sporulation in *Bacillus subtilis*. Mol Microbiol.

[47] Wittchen KD, Meinhardt F (1995). Inactivation of the major extracellular protease from *Bacillus megaterium* DSM319 by gene replacement. Appl Microbiol Biotechnol.

[48] Chen HJ, Pan SC, Shaw GC (2009). Identification and characterization of a novel intracellular poly(3-hydroxybutyrate) depolymerase from *Bacillus megaterium*. Appl Environ Microbiol.

[49] Jo SJ, Maeda M, Ooi T, Taguchi S (2006). Production system for biodegradable polyester polyhydroxybutyrate by *Corynebacterium glutamicum*. J Biosci Bioeng.

[50] Jo SJ, Matsumoto K, Leong CR, Ooi T, Taguchi S (2007). Improvement of poly(3-hydroxybutyrate) [P(3HB)] production in *Corynebacterium glutamicum* by codon optimization, point mutation and gene dosage of P(3HB) biosynthetic genes. J Biosci Bioeng.

[51] Wittchen KD, Strey J, Bultmann A, Reichenberg S, Meinhardt F (1998). Molecular characterization of the operon comprising the *spoIV* gene of *Bacillus megaterium* DSM319 and generation of a deletion mutant. J Gen Appl Microbiol.

[52] Perego M, Spiegelman GB, Hoch JA (1988). Structure of the gene for the transition-state regulator, *abrB*: regulator synthesis is controlled by the *spo0A* sporulation gene in *Bacillus subtilis*. Mol Microbiol.

[53] Phillips ZEV, Strauch MA (2002). *Bacillus subtilis* sporulation and stationary phase gene expression. Cell Mol Life Sci.

[54] Strauch M, Webb V, Spiegelman G, Hoch JA (1990). The Spo0A protein of *Bacillus subtilis* is a repressor of the *abrB* gene. Proc Natl Acad Sci USA.

[55] Strauch MA, Hoch JA (1993). Transition-state regulators: sentinels of *Bacillus subtilis* postexponential gene expression. Mol Microbiol.

[56] Levdikov VM, Blagova EV, Rawlings AE, Jameson K, Tunaley J, Hart DJ, Barak I, Wilkinson AJ (2012). Structure of the phosphatase domain of the cell fate determinant SpoIIE from *Bacillus subtilis*. J Mol Biol.

[57] Barak I, Behari J, Olmedo G, Guzman P, Brown DP, Castro E, Walker D, Westpheling J, Youngman P (1996). Structure and function of the *Bacillus* SpoIIE protein and its localization to sites of sporulation septum assembly. Mol Microbiol.

[58] Barak I, Youngman P (1996). SpoIIE mutants of *Bacillus subtilis* comprise two distinct phenotypic classes consistent with a dual functional role for the SpoIIE protein. J Bacteriol.

[59] Bradshaw N, Losick R (2015). Asymmetric division triggers cell-specific gene expression through coupled capture and stabilization of a phosphatase. Elife.

[60] Feucht A, Magnin T, Yudkin MD, Errington J (1996). Bifunctional protein required for asymmetric cell division and cell-specific transcription in *Bacillus subtilis*. Genes Dev.

[61] Bi CH, Jones SW, Hess DR, Tracy BP, Papoutsakis ET (2011). SpoIIE is necessary for asymmetric division, sporulation, and expression of sigma F, sigma E, and sigma G but does not control solvent production in *Clostridium acetobutylicum* ATCC 824. J Bacteriol.

[62] Grage K, Rehm BHA (2008). In vivo production of scFv-displaying biopolymer beads using a self-assembly-promoting fusion partner. Bioconjug Chem.

[63] Hooks DO, Rehm BHA (2015). Insights into the surface topology of polyhydroxyalkanoate synthase: self-assembly of functionalized inclusions. Appl Microbiol Biotechnol.

[64] Schaeffer P, Millet J, Aubert JP (1965). Catabolic repression of bacterial sporulation. Proc Natl Acad Sci USA.

[65] Hanahan D (1983). Studies on transformation of *Escherichia coli* with plasmids. J Mol Biol.

[66] Barg H, Malten M, Jahn M, Jahn D, Barredo J-L (2005). Protein and vitamin production in *Bacillus megaterium*. Microbial processes and products.

[67] Brandl H, Gross RA, Lenz RW, Fuller RC (1988). *Pseudomonas oleovorans* as a source of poly(beta-hydroxyalkanoates) for potential applications as biodegradable polyesters. Appl Environ Microbiol.

[68] Laemmli UK (1970). Cleavage of structural proteins during assembly of head of bacteriophage T4. Nature.

