# Supplementary material for: Engineering Bacillus megaterium for production of functional intracellular materials
Source: Microb Cell Fact. 2017 Nov 22;16:211. doi: 10.1186/s12934-017-0823-5 (PMC5700737; doi:10.1186/s12934-017-0823-5)
Supplement: Supplementary file 1 — Additional file 1. Additional tables and figures. [file 12934_2017_823_MOESM1_ESM.docx]

**Additional Tables and Figures**

**Table S1: Cultivation of PHA05 in LB with different carbon sources at 37 °C for 24 h**

| **Carbon source^a^** | **Plasmid** | **Induced (yes/no)** | **Final OD_600_^b^** |
| --- | --- | --- | --- |
| glucose | pMM-ZZCAB | no | 2.0/0.7 |
|  | pPT7-ZZCAB |  | 3.0/2.5 |
|  | p1623-ZZCAB |  | 1.8/1.4 |
| glycerol | pMM-ZZCAB | no | 2.0/0.2 |
|  | pPT7-ZZCAB |  | 2.6/1.0 |
|  | p1623-ZZCAB |  | 1.4/0.2 |

^a^ 2% final concentration
**^b^** If OD_600_ declining by final measurement, previous value is also shown.

**Table S2: Cultivation of PHA05 in A5 medium with different carbon sources at 37 °C for 24 h**

| **Carbon source^a^** | **Plasmid** | **Induced (yes/no)** | **Final OD_600_^b^** | **Induced (yes/no)^c^** | **Final OD_600_^b^** |
| --- | --- | --- | --- | --- | --- |
| glucose | pMM-ZZCAB | no | 4.0/3.6 | yes | 9.4 |
|  | pPT7-ZZCAB |  | 5.3 |  | 13.2 |
|  | p1623-ZZCAB |  | 3.4 |  | 10.9 |
| glycerol | pMM-ZZCAB | no | 2.9/1.3 | yes | 7.4 |
|  | pPT7-ZZCAB |  | 5.2 |  | 1.7 |
|  | p1623-ZZCAB |  | 2.9/1.2 |  | 8.2 |
| Fructose | pMM-ZZCAB | no | 4.0 | yes | 5.7 |
|  | pPT7-ZZCAB |  | 5.5 |  | 6.4 |
|  | p1623-ZZCAB |  | 2.9 |  | 6.3 |
| Pyruvate | pMM-ZZCAB | no | 3.5 | yes | 9.8 |
|  | pPT7-ZZCAB |  | 3.6 |  | 4.9 |
|  | p1623-ZZCAB |  | 3.5 |  | 9.2 |

^a^ 2% final concentration
**^b^** If OD_600_ declining by final measurement, previous value is also shown.
^c^ Induced at OD_600_ = 0.4-0.5 by adding 0.5 % xylose; cultures then shifted from 37 °C to 25 °C

**Table S3: Comparison of growth and PHB accumulation after 24 and 48 h of cultivation^a^**

| **Strain^b^** | **Cultivation time** | **OD_600_** | **PHB (% of CDW)^c^** |
| --- | --- | --- | --- |
| PHA05 + pPT7-CAB | 24h | 8.1 | 20.3 |
|  | 48h | 7.4 | 18.9 |
| PHA05 + pPT7-ZZCAB | 24h | 6.9 | 15.9 |
|  | 48h | 6.2 | 10.4 |

^a^ Cultivation in A5 medium supplemented with 2 % glucose at 37 °C. Induction at OD_600_ = 0.4-0.5 by addition of 0.5 % xylose and shift to 25 °C
^b^ all strains also contained pT7RNAP
^c^ PHB content determined by GC/MS

**Table S4: PHB content measured for different induction conditions^a^**

| **Xylose induction** | **PHB (% of CDW)** |
| --- | --- |
| 0.5 % xylose^b^ | 16.0 |
| 1 % xylose^b^ | 21.3 |
| repeated xylose addition^c^ | 19.8 |

^a^ Cultivation in A5 medium supplemented with 2 % glucose at 37 °C. After induction shift to 25 °C. Cultivation for 24 h.
^b^ at OD_600_ = 0.4-0.5
^c^ 0.5% IPTG at OD_600_ of 0.4-0.5 and again 4 and 8 h after initial induction

**Table S5: Oligonucleotides used in this study**

| **Oligo Name** | **Sequence** | **Source** |
| --- | --- | --- |
| ZZ-fwd-noSS | AATATTAATTAACCAAGGAGGAGGTAGGAAAATGGCACAACACGACGAAGCTGTTGATAACAAATTTAACAAAG | this study |
| ZZ-rev-BspEI | ATCCTGTCCGGAACCGTGTCTTGGAGAACTGTTAGCATCAAC | this study |
| FRT-1Fpsc | TGCCAAGCTTGCATGCCTGCAGGTCGACGAATTGGGGATCTTGAAGTT | this study |
| FRT-1Rpsc | TTTTTTGGATCCCCGCGGGTTAACAGATCTGATATCGAGTAAGTTTTTAAGCACATCAG | this study |
| FRT-2Fpsc | AAAAAAGGATCCGCTAGCGGCGCGCCGGGCCCGATATCGAATTGGGGATCTTGAAGTT | this study |
| FRT-2Rpsc | ATATGGTACCATGAGTAAGTTTTTAAGCACATCAG | this study |
| CmF-psc | ATATGAGCTCGGCGACAAACGAAAATTGGATAAAG | this study |
| CmR-psc | ATATGAGCTCCGATTGTACTGAGAGTGCAC | this study |
| SpoF-psc | ATATGCTAGCCGTTCACTGTATTTAGCGCGATG | this study |
| SpoR-psc | ATATGGGCCCCCAATTAGCAATGAACCAACAACCAG | this study |
| spo5'fwd2 | GAATAAGTATAATTAGCGGGTGGT | this study |
| spo5'fwd3 | GGTGGGAGAATGCAAAAGAC | this study |
| Cm_pMK_inner_fwd2 | AACTCTATTCAGGAATTGTCAG | this study |
| CmF | GGCGACAAACGAAAATTGGATAAAG | this study |
| M13 fwd | GTAAAACGACGGCCAGT | this study |
| spo3’rev2 | CATGATAGACGAGGAGGAGA | this study |

**
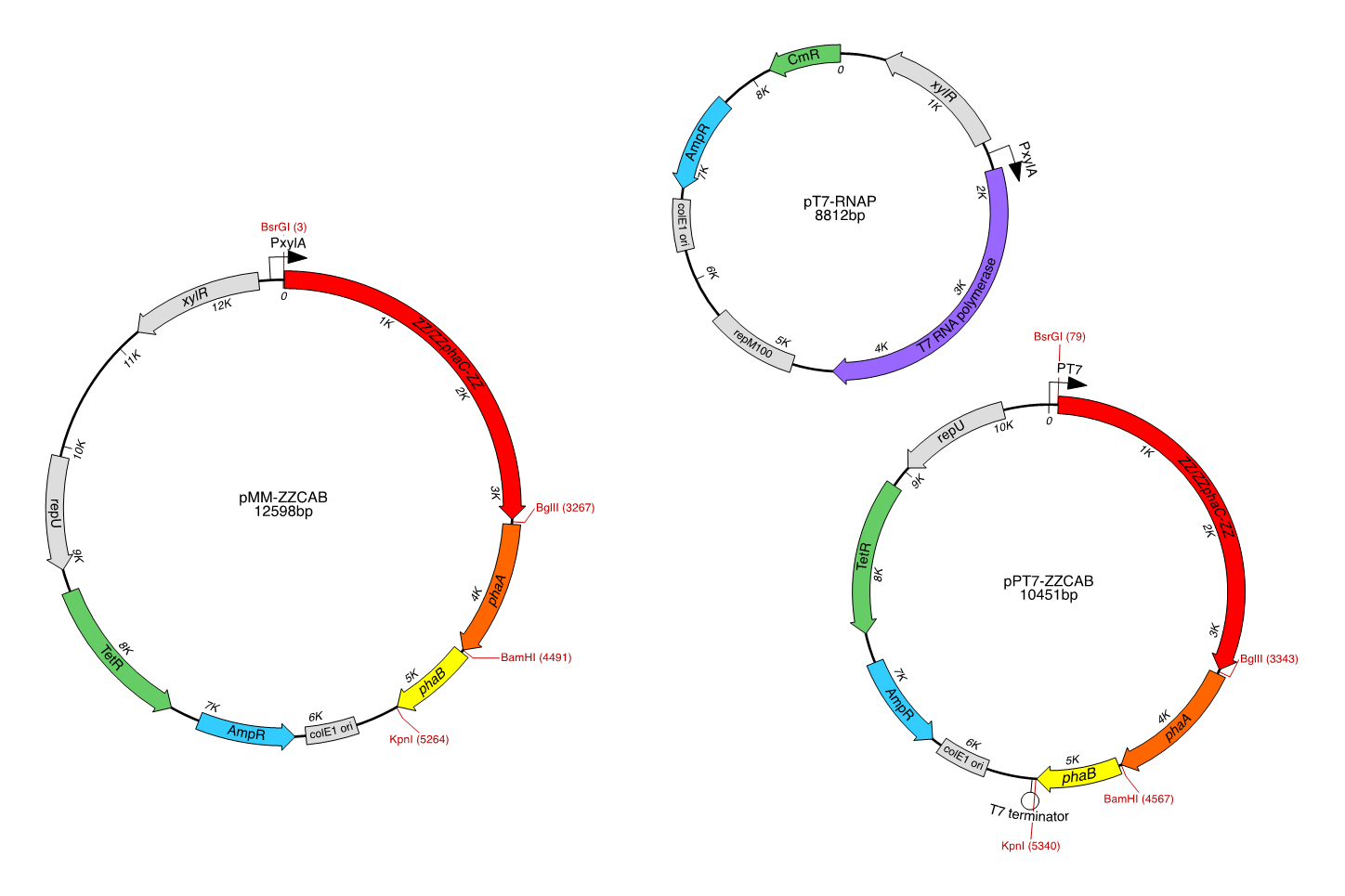
**

**Figure S1: Plasmids used for the recombinant production of Z-domain displaying PHB beads.** pMM-ZZCAB contains the gene encoding the ZZ/ZZ-PhaC-ZZ fusion protein, *phaA* and *phaB* under the control of the xylose inducible PxylA promoter. pPT7-ZZCAB contains the same genes under the control of the T7 promoter. The T7 RNA polymerase is encoded on plasmid pT7-RNAP under PxylA promoter control. Restriction sites used for cloning are indicated. P1623-ZZCAB (not shown) is identical to pMM-ZZCAB, with the exception of a modified PxylA promoter region and RBS.

**
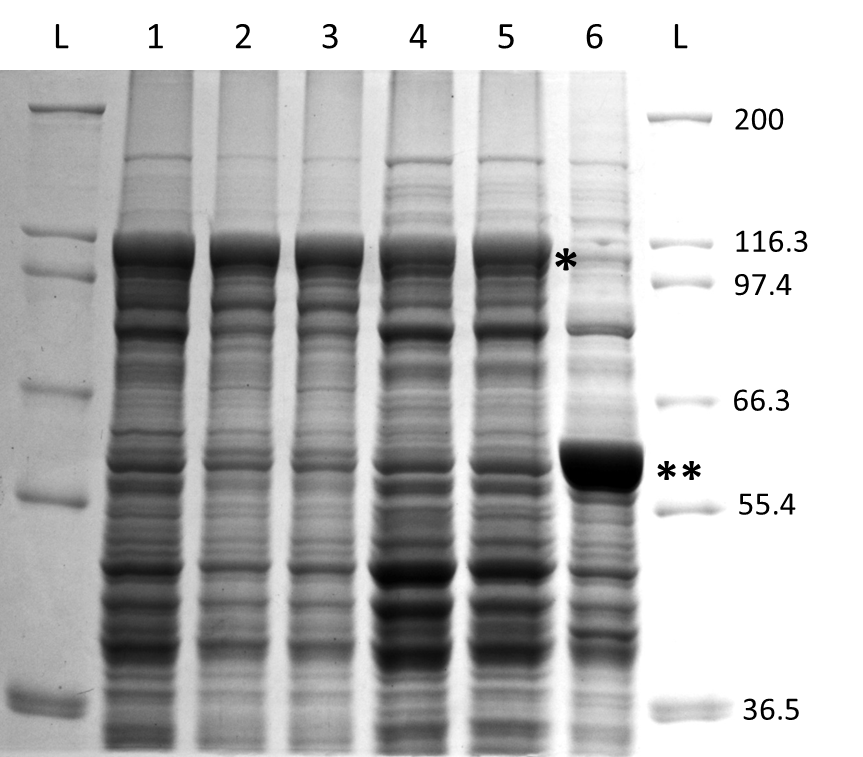
**

**Figure S2: SDS-PAGE of beads produced under different induction conditions**. Beads were isolated from *B. megaterium* PHA05 harbouring the required plasmids and cultivated in A5 medium with 2 % glucose. Lanes 1-5, ZZ-displaying beads, ZZ/ZZ-PhaC-ZZ protein with an expected molecular weight of 118 kDa (one asterisk); lane 6, wild type beads, PhaC protein with an expected molecular weight of 65 kDa (two asterisks). Equal amounts of protein as determined by Bradford were loaded in each lane. Lane 1, induced with 0.5 % xylose at OD_600_ of 0.4-0.5; lane 2, induced with 1 % xylose at OD_600_ of 0.4-0.5; lane 3, xylose added multiple times (0.5% at OD_600_ of 0.4-0.5 and same amount added again 4 and 8 h after initial induction); lane 4, induced with 0.5 % xylose at OD_600_ of 1.7; lane 5, cultivated with 3 % glucose (instead of 2 %); L, Mark 12™ protein standard (Thermo Fisher Scientific)

**
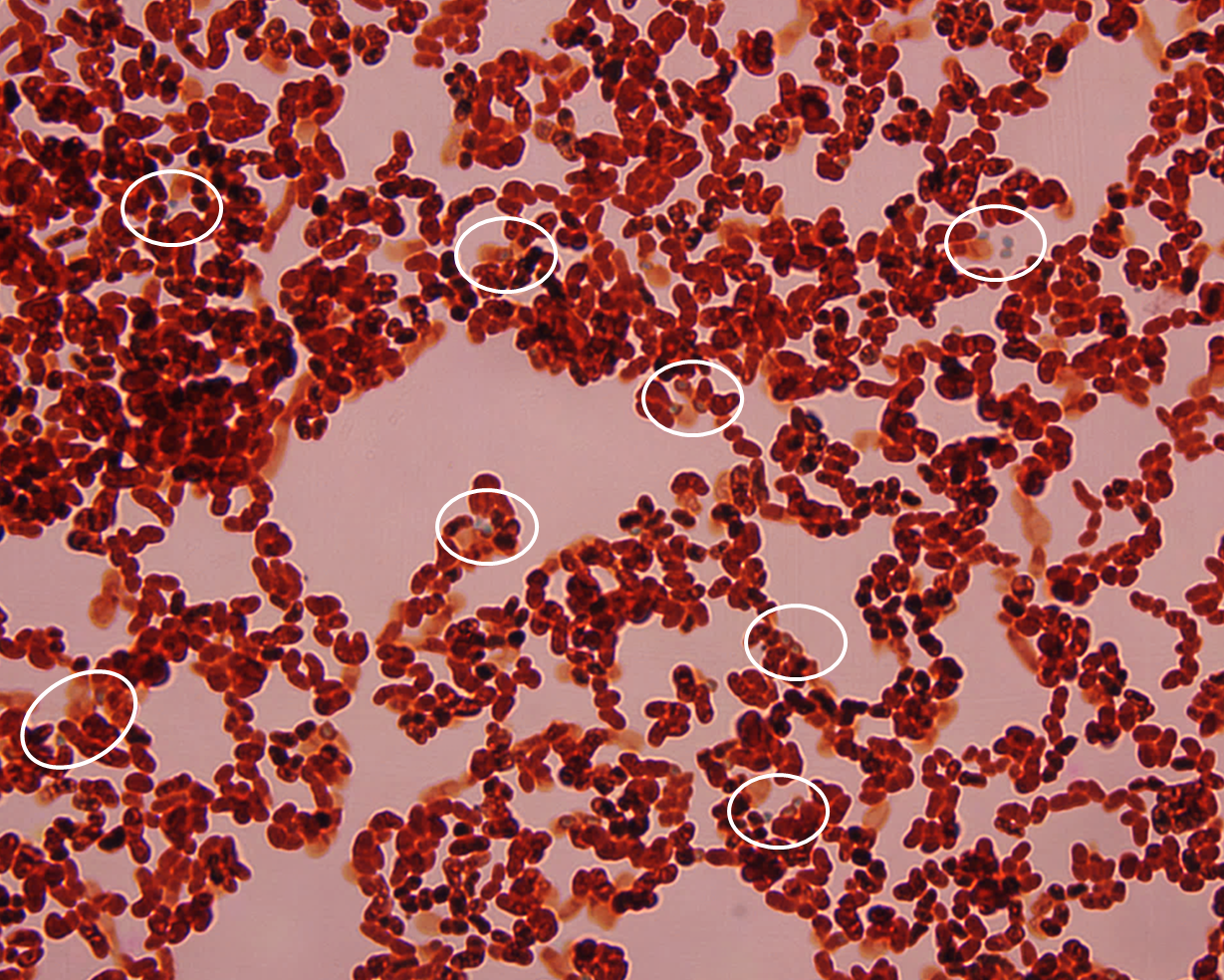
**

**Figure S3: Spore stain of *B. megaterium* PHA05 cells grown under PHB production conditions (mediated by plasmids pT7RNAP and pPT7-ZZCAB in A5 medium with 2 % glucose, induced with 1 % IPTG).** Spores are stained blue-green with malachite green, vegetative cells red with safranin. Some spore containing areas have been circled in white.
